# A cross-sectional study to determine impact of social media influencing patient choices in aesthetic dentistry

**DOI:** 10.6026/973206300212527

**Published:** 2025-08-31

**Authors:** Shaista Haleem

**Affiliations:** 1 Department of Aesthetic and Restorative Dentistry, College of Applied Medical Sciences, Riyadh Elm University, Riyadh-Kingdom Saudi Arabia

**Keywords:** Aesthetic dentistry, social media, patient preference, dental marketing, peer influence, Saudi Arabia, digital health behaviour

## Abstract

The effect of social media exposure on adult patient choices in aesthetic dentistry in Saudi Arabia is of interest. An online structured and validated questionnaire was applied to 335 individuals and it was found that social media use, peer impact and visual content had a significant role in the choice of dental clinic. Although 72.8% of the participants involved preferred social media as their primary channel of information, trust was derived more from personal or family experience when compared to celebrity testimonies. However, statistical analysis did not find the relation between the social media-affected choices and sex and age. The results highlight the importance of digital interaction in aesthetic dental preferences in today's society.

## Background:

With the evolving awareness, aesthetic dental procedures have seen an exponential rise in demand in recent past all across the globe. Dentistry, classically oriented to diagnosing and treating oral diseases, has recently evolved to provide services aimed at improving facial and dental aesthetics, including veneering, bleaching, orthodontics and smile makeover [1]. This balance reflects beyond the world of fashion, in a society that is increasingly obsessed with appearance and how we look at ourselves, under the influence of digital and media systems [2]. Social media has revolutionized how people are finding health information and dentistry is no exception. With the number of global internet users surpassing 4.5 billion and global active social media users reaching more than 3.8 billion, platforms such as Instagram (Meta Platforms Inc), YouTube (Google) and TikTok (TikTok Inc) have become powerful ways that patients come into contact with cosmetic dental services [3]. In Saudi Arabia, the penetration and engagement levels are very high, around more than 25 million people use social media in the country, which refers to that around 72% of population using social media [4]. Social media is now not only just a means of marketing and communication and is influencing patient expectations and decisions. It has been reported that visual depictions (including before-and-after imagery and endorsements made by celebrity figures), in particular, can impact beliefs about the desiredness and success of treatment [5]. In addition, younger adults and females have been found to be receptive to using social media to select providers of cosmetic dental care [6]. Tariq *et al.* (2024) conducted a cross-sectional study to explore the role of social media in shaping public perception of dentofacial aesthetics. The authors found that frequent exposure to aesthetic dentistry content on platforms such as Instagram, Facebook, and TikTok significantly influenced how individuals evaluated dental appearance and determined treatment needs. The study emphasized that social media not only raises awareness of various cosmetic dental procedures but also impacts patient expectations, often creating idealized standards of dental beauty. These findings underline the growing importance for dental practitioners to understand the influence of digital platforms on patient choices, enabling them to provide realistic treatment options and manage expectations effectively [7]. Therefore, it is of interest to examine the role of social media in shaping aesthetic dental care choices in Saudi Arabia, particularly as visual influence continues to grow among younger demographics.

## Methodology:

This cross-sectional study was conducted to assess the influence of social media on patient choices regarding aesthetic dentistry among the adult population of Saudi Arabia. The study protocol received approval from the Institutional Review Board (IRB) of Riyadh Elm University with the reference number FUGRP/2023/310/952/852. The subjects were adult SNS users aged 19 to 60 years having received or considered receiving aesthetic dental treatment and high-end treatments such as orthodontics, tooth whitening, anterior restorations, veneers, Hollywood smiles and cosmetic contouring. The following were excluded from the study population: ages less than 19 aged more than 60, non-users of social network media, who had never thought about aesthetic dental treatment. The sample size was determined by an online sample size calculator (www. calculator. net) according to the entire adult population of Saudi Arabia 95% confidence level and 5% margin of error. The survey was completed by 335 people. Data were collected using a standardized, pretested self-administered questionnaire prepared in Arabic, which is the native language of the study participants. The questionnaire was a modified version of the RSES and items that have been elsewhere employed in the study Al Awdah *et al.* [8] and Binalrimal *et al.* [9] with necessary adjustments according to the situation of the present study. It comprised 28 questions in closed- and open-ended formats related to demographic characteristics, social media use, trust in online health information and the impact of digital content (ads, before/after results and celebrity endorsements) in the decision of choosing dental services. The questionnaire was pilot tested with dentists in the Department of Prosthodontics to assess clarity and content validity prior to the deployment. Using their comments, necessary amendments were made. The refined questionnaire was uploaded on Google Forms and shared across social media link: WhatsApp, Twitter and Instagram. Participation was optional and respondents implied consent by responding to the online form. At the start of the survey, a brief note explaining the aim of the study and assurance regarding anonymity was given. This trial was conducted in accordance with the Declaration of Helsinki ethical standards. Personal identifiers were not used and all responses were maintained as strictly confidential. All data in both types: hard and soft copy was kept private at Riyadh Elm University and made confidential between the research team members. The data were analyzed using IBM SPSS Statistical software for Windows version 25.0. The frequency and percentage of demographic and response patterns were described using descriptive statistics. To examine the relationships between socio-demographic aspects and social media impact on aesthetic dental decisions, inferential analysis by means of Chi-square tests were performed. A p-value 0.05 was considered statistically significant, at a 95% confidence interval.

## Results:

The socio-demographic characteristics of the study participants (N = 335) are shown in Table 1 (see PDF). The largest age group was 18-25 years (50.1%), followed by 26-35 years (30.4%). Participants aged 36-45 years accounted for 10.1%, those aged 46-54 years for 7.2% and only 0.9% was above 55 years. A small proportion (1.2%) was younger than 18 years. The sample included more females (64.2%) than males (35.8%). Overall, 56.1% of participants were employed and 43.9% were unemployed. Table 2 (see PDF) shows participants' social media usage related to dental care. A majority (59.7%) followed dentists or dental clinics on social media. Most participants (89.6%) preferred dental information from social media over traditional media. The figure 1 (see PDF) shows factors influencing patient choices in aesthetic dentistry. Personal or family experience is the most significant factor with a frequency of 160. Comments and opinions follow with 115, indicating their importance in decision-making. Pictures hold less influence, with a frequency of 33. The number of followers has the least impact, with a frequency of 25. The table 3 (see PDF) highlights the influence of social media on dental clinic selection. A majority of respondents (64.2%) reported that a dentist's social media activity influenced their decision, while 35.8% said it did not. About 31.9% of people shared their dental visit experiences on social media, while 68.1% did not. Criticism on social media had a significant impact on clinic choice, with 83.0% acknowledging its effect. Lastly, 72.8% of respondents stated they would visit a clinic after viewing its social media page, while 27.2% disagreed. The figure 2 (see PDF) illustrates the responses to a particular question. A total of 33.7% of respondents answered "Yes," while the majority, 66.3%, answered "No." This shows a clear preference or majority against the statement represented by the chart. Similarly, 82.4% agreed that reading criticism about a dentist on social media would change their opinion. Furthermore, 72.8% believed that viewing a clinic's social media page could encourage a visit. Regarding endorsements, Table 4 (see PDF) shows that 66.6% of respondents were influenced by before-and-after treatment images on social media. Conversely, 33.4% were not influenced by such visuals. Celebrity endorsements had less impact. Only 37.3% of participants said they would consider a clinic promoted by a celebrity. The remaining 62.7% were not influenced. Peer influence was stronger. About 83.0% reported they would inquire about a dentist or clinic if a friend or family member had undergone aesthetic treatment, whereas 17.0% would not. Table 5 (see PDF) shows the association between gender and social media as the first choice for dental aesthetic information. Among females, 54 chose social media as not the first choice, while 160 chose it as their first choice. Among males, 36 chose it as not the first choice, and 84 chose it as the first choice. The Chi-square value is 1.448 with a p-value of 0.485, indicating no significant association between gender and the preference for social media as the first choice for dental aesthetic information. Table 6 (see PDF) presents the association between age and the selection of a dentist based on celebrity advertisements. Among individuals under 18 years, none chose a dentist based on advertisements, while 4 did. In the 18-25 years age group, 122 did not choose based on advertisement, while 45 did. For the 26-35 years group, 76 did not choose, and 26 chose based on advertisement. In the 36-45 years group, 23 did not choose, and 11 did. The 46-54 years group had 19 who did not choose, and 5 who did, while those above 55 years had 2 who did not choose, and 1 who did. The Chi-square value is 3.588 with a p-value of 0.964, indicating no significant association between age and the likelihood of choosing a dentist based on celebrity advertisements.

## Discussion:

This study investigated how social media influences patient decisions regarding aesthetic dental treatments among adults in Saudi Arabia. The findings suggest a strong and growing role of digital platforms in shaping public preferences and choices in the field of cosmetic dentistry. The age profile indicated that the majority of respondents were youth, mostly 18-35 years old and over the half of them were women. This corresponds to previous findings suggesting that younger generations and females are more receptive to aesthetic augmentations and more involved in visual social media platforms, such as Instagram and Snapchat [10]. The over-representation of feminine responders could also be associated with the female curiosity about appearance-related services as reported from the past gender-specific aesthetic literature [11]. The majority of participants (59.7%) followed dental professionals or dental clinics in social media and a significant majority (89.6%) preferred dentists to communicate with them through social media over traditional methods of communication. In addition, 72.8% that the first source of information they considered when thinking about aesthetic dental treatment was social media. This is consistent with global trends, where we find ourselves becoming increasingly reliant on social platforms for health information and patients are using it as an effective means of archiving health information [12]. This paradigm shift highlights that there is a niche for the dental professional to create an open and trustworthy online presence to ethically and appropriately manage patient expectations. Interestingly, although 51.3% of respondents expressed trust in the information they found on social media, trust was driven more by personal/family history (47.8%) and comments made by peers (34.3%) rather than number of followers or testimonial images. This mirrors the shift from celebrity to peer-based influence as a more convincing form of social proof that has been observed in other health behavior domains [13]. With respect to behavioral impact, 64.2% reported that a dentist's SM activity had impacted their decision to attend the clinic and 72.8% would consider visiting a clinic after viewing its SM page. Indeed, 83.0% said that negative criticism on social media would dissuade them from attending a dentist. These findings underscore the importance of online reputation management and indicate that social media-based reviews and patient feedback indeed can have a major influence on clinical footfall, consistent with earlier digital marketing research in health care [14].

Visuals were especially effective as 66.6% of respondents said before-after photos on social media had a significant impact on their dentist selection. But the influence of celebrities paled in comparison. Only 37.3% would give priority to clinics promoted by celebrities and 26.3% would select a dentist advertised by a celebrity. These findings reflect a movement from celebrity-based decision making to authenticity, peer referral and disclosure of results. This is consistent with recent literature that indicates influencer fatigue has created a patient base that demands authenticity over ad [15]. Furthermore, although examples of esthetic dental treatment advertised with special offers on social media attracted one-third of the survey participants (n=586, 33.7%); the non-promotional factors such as quality of treatment had even more preference for esthetic treatment (66.3%). This discovery reinforces the need to develop patient trust through professional recomendation rather than offering discounts and leveling down the perceived quality of the service [16]. Statistical analysis showed no associations emerged between gender and preference for social media as the first source if information or between age and influence of celebrity endorsement. These findings imply that the effects of social media on elective aesthetic dental decision-making is fairly uniform across sexes and age- perhaps, as a reflection of ubiquitous and integrated place of digital media as part of daily health related decision-making process. Although the study provides important information about the online behaviors of adult dental patients in Saudi Arabia, it is not without challenges. This was a self-report, online survey study and may have been affected by response bias and a lack of representativeness of non-social media users. Furthermore, the use of a cross-sectional design does not allow drawing casual conclusion. Negative association the influence of social media on patient satisfaction and experience and clinical outcomes over time longitudinal research is needed to evaluate the effects of social media on patient satisfaction and experience and clinical outcomes.

## Conclusion:

We show that social media significantly influences patient preferences and decision-making in aesthetic dentistry, particularly through peer experiences, visual content and online engagement by dental professionals. While celebrity endorsements had limited impact, trust was primarily guided by personal or family recommendations and authentic patient feedback. These findings highlight the evolving dynamics of patient behavior in the digital age and underscore the importance of ethical, evidence-based social media communication in dental practice.
